# Supplementary material for: Body Composition of Infants Born with Intrauterine Growth Restriction: A Systematic Review and Meta-Analysis
Source: Nutrients. 2022 Mar 4;14(5):1085. doi: 10.3390/nu14051085 (PMC8912478; doi:10.3390/nu14051085)
Supplement: Supplementary file 1 [file nutrients-14-01085-s001.zip › nutrients-1606354-supplementary.pdf]

nutrients MDPI

SUPPLEMENTARY MATERIAL

Supplementary Table S1. Search Strategy

Databases

- Medline
- Web of Science
- Scopus
- EMBASE
- LILACS

Medline (2009-2021)

Concept	Mesh terms	Text words
IUGR	Fetal growth retardation	Fetal Growth<br>Intrauterine Growth<br>IUGR<br>Fetal growth retardation<br>Fetal growth restriction<br>Fetal nutrition disorders<br>Growth abnormalit* AND (fetus OR fetal)<br>growth-restricted<br>growth restricted fet*<br>(fetus OR fetal) AND retard*[tw]<br>Infant, Low Birth Weight<br>infant, small for gestational age<br>SGA
Body composition	Body composition	Body composition<br>Composition, Body<br>Compositions, Body<br>Distribution, Body Fat<br>Fat Distribution, Body<br>Body Fat Patterning<br>Fat Patterning, Body<br>Patterning, Body Fat<br>Body fat<br>Adipos*<br>Prenatal Doppler<br>Fetal Nutrition Disorder<br>Nutrition Disorder, Fetal<br>Nutrition Disorders, Fetal<br>Fetal Malnutrition<br>Malnutrition, Fetal<br>Catch-up fat<br>Adiposity<br>Lean mass<br>Fat-free mass<br>Ponderal index<br>Fat mass index
Infant	Infant	Infant

		Low-Birth-Weight Infant<br>Infant, Low-Birth-Weight<br>Infants, Low-Birth-Weight<br>Low Birth Weight Infant<br>Low-Birth-Weight Infants<br>Low Birth Weight<br>Birth Weight, Low<br>Birth Weights, Low<br>Low Birth Weights<br>Infant, Newborn<br>Infant, Premature<br>Infant, Preterm

Search Strategy (dated 10 August 2021)

IUGR		Body composition		Infant
Fetal growth restriction[Mesh] OR Fetal Growth[Tw] OR Intrauterine Growth[tiab] OR IUGR[tiab] OR Fetal growth retardation[Tw] OR Fetal growth restriction[tiab] OR (Growth abnormalit* AND (fetus OR fetal)) OR Growth-restricted[tiab] OR Growth restricted fet*[tiab] OR ((fetus OR fetal) AND retard*[tw]) OR Infant, Low Birth Weight[tiab] OR infant, small for gestational age[tiab] OR sga[tiab] OR Fetal Development[Tw] OR Fetal Nutrition Disorder*[Tw] OR Nutrition Disorder, Fetal[Tiab] OR Nutrition Disorders, Fetal[Tiab] OR Fetal Malnutrition[Tiab] OR Malnutrition, Fetal[Tiab] OR ("preeclampsia"[Title/Abstract])	AND	Body composition[Mesh] OR Body composition[Tw] OR Composition, Body[tiab] OR Compositions, Body[tiab] OR Distribution, Body Fat[tiab] OR Fat Distribution, Body[tiab] OR Body Fat Patterning[tiab] OR Fat Patterning, Body[tiab] OR Patterning, Body Fat[tiab] OR Adipos*[tiab] OR Fetal Development[Tw] OR body fat[Tw] OR Prenatal doppler[Tiab] OR catch-up fat[Tiab] OR "lean mass"[Text Word] OR "fat free mass"[Text Word] OR "fat mass index"[Text Word]	AND	infant*[Tw] OR child*[Tw] OR Infant, Newborn[Tw] OR neonat*[Tw] Low-Birth-Weight Infant[Tw] OR Infant, Low-Birth-Weight[Tw] OR Infants, Low-Birth-Weight[Tw] OR Low Birth Weight Infant[Tw] OR Low-Birth-Weight Infants[Tw] OR Low Birth Weight[Tw] OR Birth Weight, Low[Tw] OR Birth Weights, Low[Tw] OR Low Birth Weights[Tw] OR Infant, Newborn[Tw] OR Infant, Premature[Tw] OR Preterm[Tw]

Web of Science

#1 TS= Fetal growth restriction OR Fetal Growth OR Intrauterine Growth OR IUGR OR Fetal growth retardation OR Fetal growth restriction OR Growth restricted fet* OR intrauterine growth restriction

#2 TS=(OR body composition OR (infant OR child) or or

#3 TS= (adipos* adj2 fatness) OR body composition OR body fat distribution OR body fat Patterning OR catch-up fat OR lean mass OR fat free mass OR fat mass index

#4 TS=(infan* adj2 child*) OR newborn OR neonat* OR Low-Birth-Weight Infant

#1 AND #2 AND #3 AND #4 Indexes = 17

Scopus

((TITLE-ABS-KEY (Fetal growth restriction OR Fetal Growth OR Intrauterine Growth OR IUGR OR Growth abnormalit* OR Growth restricted fet*[tiab] OR fetus retard* OR Low Birth Weight OR small for gestational age OR sga) AND (TITLE-ABS-KEY ((body compos* OR Fat distribut* OR Body Fat Pattern* OR Adipos* OR Fetal Development OR catch-up fat OR lean mass OR fat free mass) AND (TITLE-ABS-KEY (infant* OR child* OR newborn OR neonat* OR low-Birth-Weight OR premature OR Preterm))

CINAHL

(((Fetal growth restriction OR "IUGR").ti,ab OR “SGA” OR small for gestation* OR AND ((body composition OR fat free mass OR fat mass OR adipos*).ti,ab) AND (child* OR infant OR toddler))

Supplementary Table S2. Excluded studies

Aneesh M, 2019	Anthropometry based BC
Aurensanz Clemente, 2003	Article in Spanish
Ay Land, 2009	Separate data for SGA not given
Beltrand 2009	Anthropometry based BC
Bortolotto CC 2021	Adult composition
Breij LM 2015	healthy newborns
Catalano PM 1992	Anthropometry based BC
Crume TL, 2016	Separate data for SGA not given
Darendeliler F 2008	Adult composition
De Curtis M 2002	Preterm
Fonseca MJ 2015	Anthropometry based BC
Goldenberg RL 1997	Review article
Grandi C 2021	Anthropometry based BC
Hartnol 2000	Dilution method (early 2000)
Hediger ML 1998	Anthropometry based BC
Hernandez MI 2012	BC assessed t 24 months
Kotecha SJ 2018	Review article
Kramer M S 2014	healthy newborns
Krochik AG 2010	Spanish article
Lain 2006	Fetal BC
Lapillonne A 1997	before 2000
Larciprete G 2005	Fetal BC
Lee w, 2009	Separate data for SGA not given
Martínez-Aguayo A 2007	Anthropometry based BC
Meas T 2008	Anthropometry based BC
Njuieyon F 2009	Anthropometry based BC
Okada T 2015	Review
Petersen S 1992	Anthropometry based BC
Resende CB 2021	Adult/ BIA
Santos S 2016	Anthropometry based BC
Sayer AA 2005	Review article
Vd Wagen 1986	Dilution technique - old method
Wiechers C 2019	healthy newborns
Willemsen RH 2007	Anthropometry based BC

BC- body compoition, SGA- small-for-gestational age

Supplementary Table S3. Risk of bias of the included studies

Study ID	Selection			Comparability	Outcome		Total
	Representativeness of the exposed cohort (*)	Selection: non exposed cohort(*)	Ascertainment of exposure(*)	Comparability of groups on basis of design or analysis (**)	Outcome: assessment(*)	Adequacy of follow-up(*)	8⋆
de Zegher F, 2012	-	*	*	**	*		5*
Demarini S, 2006	-	*	*	**	*		5*
Giannì M, 2016	-	*	*	-	*		3*
Koo W, 2004	-	*	*	**	*		5*
Law TL, 2011	-	*	*	**	*		5*
Law TL, 2012	-	*	*	**	*		5*
Ibañez L, 2008	-	*	*	-	*		3*
Ibañez L, 2010	-	*	*	**	*		5*
Modi N, 2006	-	*	*	**	*		5*
Mazarico R, 2016	-	*	*	**	*	*	6*
Moyer-Mileur H, 2009	-	*	*	**	*		5*
Schmelzle H, 2007	-	-	*	-	*		2*
van de Langemaat M, 2014	-	*	*	**	*	*	6*
Verkauskiene R, 2007	-	*	*	**	*		5*
Villela L, 2018	-	*	*	**	*	*	6*
Kuriyan R, 2020	-	*	*	**	*		5*
Larsson A, 2019	-	*	*	**	*	*	6*
Roggero P, 2011	-	*	*	**	*	*	6*

Supplementary Figure S1

Secondary outcomes

Figure 1. Forest plot showing mean differences, between IUGR/SGA and normal intrauterine growth / AGA infants, in 1a) weight at birth - preterm infants; 1b) weight at birth - term infants; 1c) length at birth - preterm infants; 1d) length at birth - term infants; 1e) head circumference at birth - preterm infants; and 1f) head circumference at birth - term infants.

1a) Preterm infants at birth

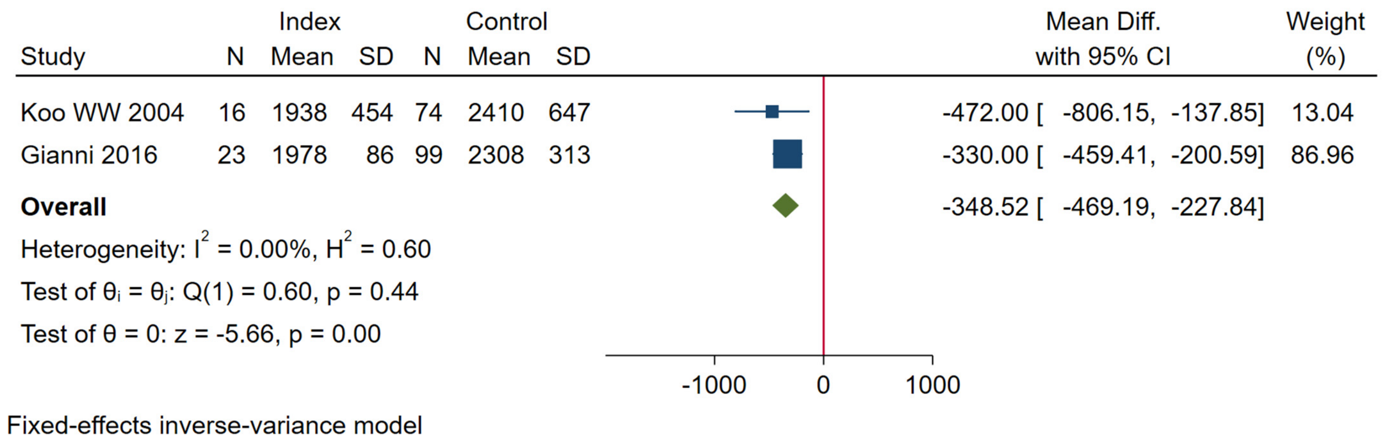

1b) Term infants

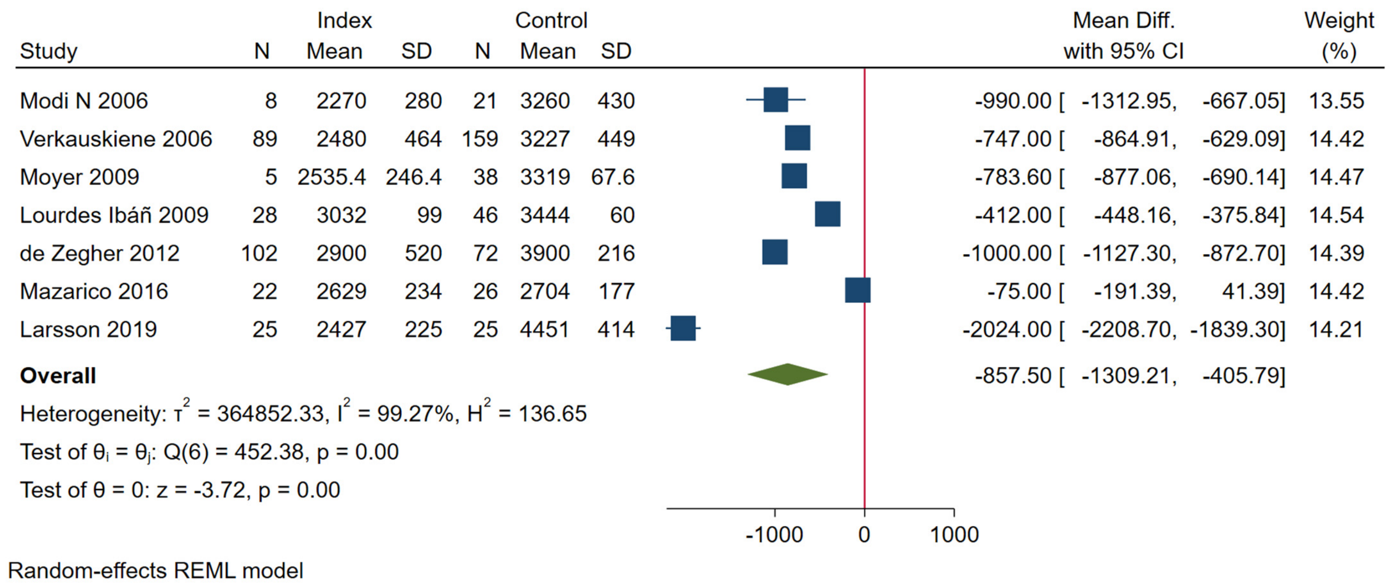

1c) Preterm infants

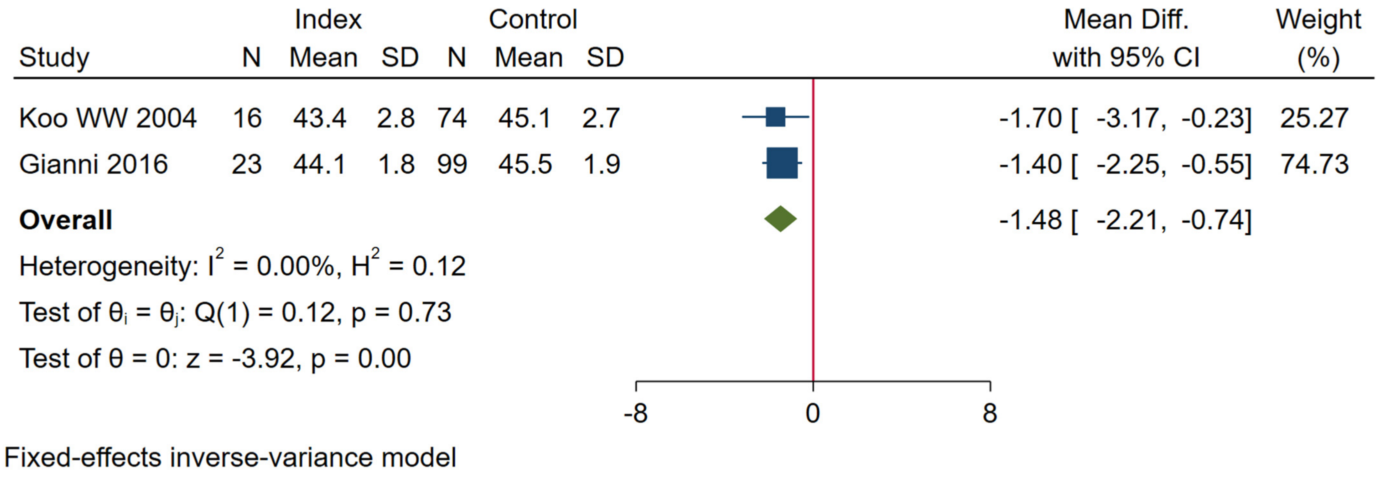

Id) Term infants

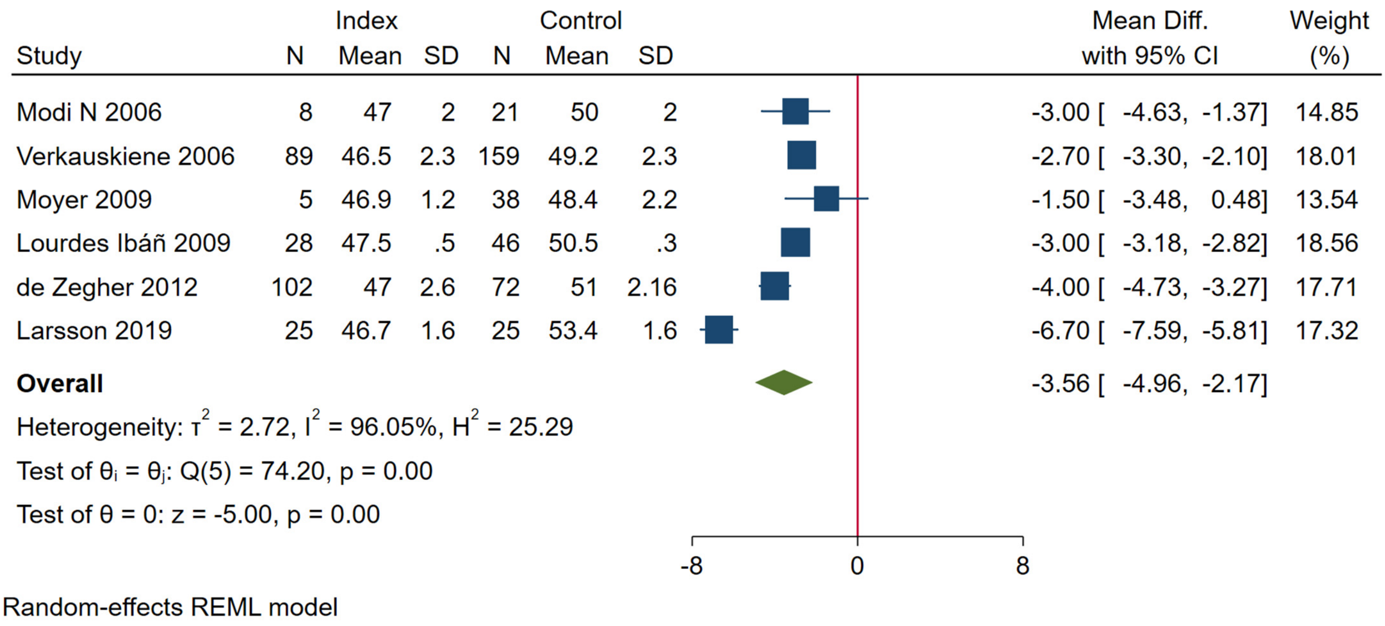

1e) Preterm infants

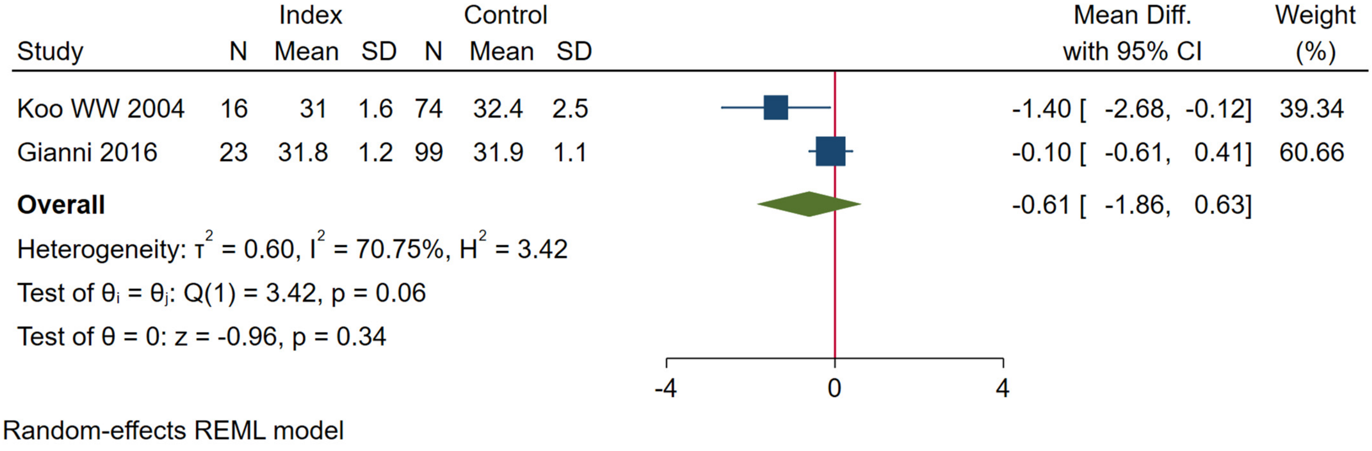

1f) Term infants

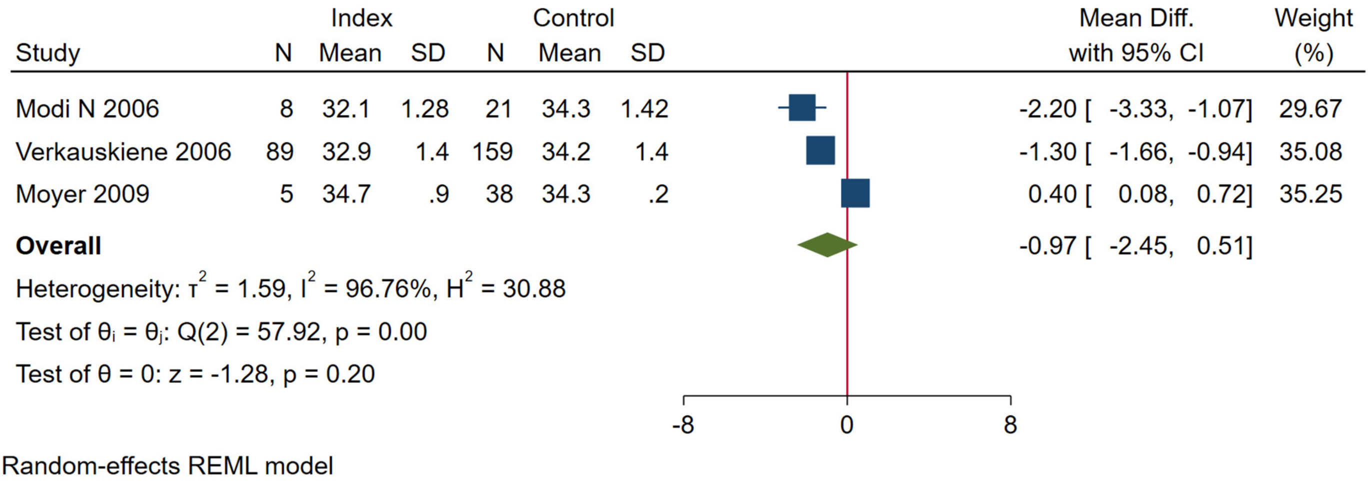

Financial Disclosure: The authors declare they have no financial relationships relevant to this article to disclose.
Funding: No specific funding was received for this review.
Conflict of Interest: Authors declare no conflict of interest relevant to this review.
